# Prevalence and factors influencing overweight/obesity and poor vision in children and adolescents before and after the COVID-19 pandemic in a city in Sichuan Province

**DOI:** 10.3389/fpubh.2025.1582864

**Published:** 2025-05-21

**Authors:** Shian Liu, Nana Li, Nian Liu, Tianyu Fu, Yongkang Mao, Jingchang Du, Yanfeng Zhu

**Affiliations:** ^1^Mianyang Center for Disease Control and Prevention, Mianyang, China; ^2^School of Public Health, Chengdu Medical College, Chengdu, China; ^3^Changping District Center for Disease Control and Prevention, Beijing, China

**Keywords:** children, adolescents, overweight, obesity, poor vision, common diseases

## Abstract

**Background:**

Overweight/obesity and poor vision are currently common health problems for children and adolescents, and the change in overweight/obesity and poor vision in children and adolescents after the COVID-19 pandemic is unclear. The aim of this study was to investigate the changes in the prevalence of overweight/obesity and poor vision in children and adolescents before and after the COVID-19 pandemic, along with the influencing factors.

**Methods:**

A survey was conducted from 2019 to 2021, involving a cohort of 33,158 children and adolescents for the purpose of gathering their general demographic information and administering questionnaires. *χ*^2^ test was employed to compare the prevalence of overweight/obesity and poor vision across various demographic characteristics, while binary logistic regression was utilized to assess the statistical significance of correlation factors.

**Results:**

The prevalence of overweight/obesity was 25.43% in 2019, increasing to 31.28% in 2020 with the COVID-19 pandemic, and falling back to 24.64% in 2021. At the same time, the prevalence of poor vision was 68.02% in 2019, decreased to 61.30% in 2020, and recovered to 72.18% in 2021. Additionally, regression analysis revealed associations between overweight/obesity and factors such as gender, place of residence, frequency of fruit consumption, smoking status, moderate to high-intensity exercise and outdoor activity time. Factors associated with poor vision included gender, place of residence, eye exercise frequency, location of recess activities, turn off the lights when looking at electronic screens after dark, reading books or electronic screens while lying down, reading books or electronic screens when walking or riding in a car, outdoor activity time, and parental myopia.

**Conclusion:**

The COVID-19 pandemic has had profound implications for the physical and mental well-being of children and adolescents, resulting in noticeable fluctuations in rates of overweight/obesity and poor vision before and after the outbreak of the pandemic. Thus, in the widespread implementation of interventions such as home isolation, school closures, and extensive use of the internet, prioritizing the health of adolescents, timely policy adjustments, and specific preventive actions are vital in avoiding such occurrences.

## Introduction

In December 2019, the outbreak of COVID-19 began in China and rapidly spread globally (1). To curb the transmission of the virus, the Chinese government mandated the closure of schools across the country in early 2020, suspending in-person classes and shifting to online education (2). It is estimated that more than 220 million school-aged children and adolescents were confined to their homes (3). However, measures such as home quarantine and school closures, while effectively preventing the spread of COVID-19, may also indirectly have a detrimental impact on child health and development (4, 5).

During lockdown and quarantine measures in response to the COVID-19 pandemic, children and adolescents experience reduced outdoor activity time and increased screen time and sedentary behavior, which can lead to adverse health outcomes including weight gain, increased myopia, and psychological problems. Numerous international studies have reported significant health challenges faced by children and adolescents during the COVID-19 pandemic, particularly concerning increases in overweight, obesity, and poor vision. For instance, the prevalence of childhood obesity in Massachusetts rose from 15.1% in 2018 and 15.7% in 2019 to 17.3% in 2020 (6). Similarly, in the Philadelphia area of Pennsylvania, the prevalence of childhood obesity increased from 13.7% in 2019 to 15.4% in 2020 (7). Additionally, researchers from Italy and India have noted a marked increase in cases of myopia among children following the imposition of COVID-19 restrictions (8, 9). These studies highlight negative changes in the health status of children and adolescents abroad compared to before the pandemic. However, it remains unclear how health problems such as overweight/obesity and poor vision among Chinese children and adolescents have changed before versus after the COVID-19 pandemic. To our knowledge, most studies conducted within China only focus on specific health issues like poor vision, overweight or obesity without comprehensive monitoring of common diseases or factors influencing them among this population prior to and post COVID-19 outbreak. Consequently, there is a pressing need to enhance scientific research in this domain.

This study aims to assess the prevalence of overweight/obesity and poor vision, along with their associated factors, among primary and secondary school students in a city within Sichuan Province from 2019 to 2021. The findings are intended to inform improvements in the health and well-being of children and adolescents and guide the implementation of targeted interventions.

## Materials and methods

### Study design and participants

Participants of this study were from the “2020 National Student Common Diseases and Health Factors Surveillance and Intervention Project.” The project aims to monitor major common conditions like myopia and obesity in children and adolescents, as well as their key health-related factors. Using a random stratified cluster sampling method, one urban district and one rural county were selected in Mianyang City, Sichuan Province, covering a total of 12 schools. Random cluster sampling was performed at the class level, with a minimum of 80 students per grade. Through questionnaires, physical examinations, and other methods, data on basic demographics, lifestyle, and health status were collected from 33,394 students. After excluding 236 cases (0.7%) with missing key information such as age, gender, and outcome indicators, a final total of 33,158 children and adolescents aged 6–18 from 2019 to 2021 were included as valid subjects for analysis.

### Measurements

Standardized equipment was used to measure the weight and height of the participants, who were dressed in lightweight clothing and barefoot. Body mass index (BMI) was calculated using the formula: weight (kg)/height (m)^2^. Overweight and obesity were classified according to the criteria set forth in the “Screening for Overweight and Obesity in School-Age Children and Adolescents” (WS/T 586–2018). Poor vision was assessed using a standard logarithmic visual acuity chart. Participants sat at a distance of 5 meters. The left eye was covered with an eye shield to assess the vision of the right eye, followed by covering the right eye to evaluate the vision of the left eye. Uncorrected visual acuity below 5.0 was classified as poor vision. In cases of unequal visual acuity between the two eyes, the lower value is used for statistics.

### Quality control

This study enforced comprehensive quality control measures to ensure data and result quality and mitigate errors and biases. When dealing with missing data, 236 children with missing key demographic information were excluded. For other variables with missing data (missingness <5%), we used mean substitution for normally distributed data and median substitution for significantly skewed data. When selecting samples, both urban and rural areas were considered. Random cluster sampling by grade was conducted to ensure equal opportunities for student participation and minimize selection bias. Before data collection, all staff received unified training on the use of standardized measurement equipment and questionnaire completion to ensure data accuracy and consistency, and to reduce information bias. During data processing, we thoroughly checked data completeness and logical consistency, verified and corrected anomalous data on weight, height, visual acuity, etc.

### Statistical analysis

Data analysis was performed using SPSS version 26.0. Descriptive statistics were presented as case numbers and percentages. The *χ*^2^ test was applied to assess differences between groups. After identifying statistically significant factors through univariate analysis of variance, binary logistic regression was employed to investigate the factors associated with overweight/obesity and poor vision. All *p*-values were two-sided, with statistical significance set at *p* < 0.05.

### Ethics statement

The data utilized in this study was sourced from the non-public database of Mianyang Center for Disease Control and Prevention, before the study started, we had obtained the consent for data use and signed a confidentiality agreement with the data manager. All data used has been de-identified and used solely for the purpose of this study. All research procedures included in this study adhered with the Declaration of Helsinki. Written informed consent to participate in this study was provided by the participants’ legal guardian/next of kin.

## Results

### Socio-demographic characteristics

A total of 33,158 students were included in the study, with 8,489 enrolled in 2019 (boys, 50.96%; girls, 49.04%) at a mean age of 11.20 ± 3.35 years, predominantly from urban areas (69.98%). In 2020, the cohort comprised 8,025 students (boys, 51.54%; girls, 48.46%) with a mean age of 11.80 ± 3.40 years, where a majority (70.78%) also resided in urban areas. The 2021 enrollment included 16,644 students (boys, 52.74%; girls, 47.26%), with an average age of 12.43 ± 3.35 years, most residing in rural areas (56.03%) (Table 1).

**Table 1:** The distribution of populations with different demographic parameters.

Categories	2019	2020	2021
Gender			
Male	4,326	4,136	8,778
Female	4,163	3,889	7,866
Age			
6–8	1800	1709	3,025
9–12	3,045	2,805	5,152
13–15	2,190	2,121	4,078
16–18	1,454	1,390	4,389
Grade			
Primary school	4,376	4,201	7,668
Junior school	2,366	2,233	3,774
Senior school	1,426	1,355	2,887
Vocational high school	321	236	2,315
Region			
Urban	5,941	5,680	7,319
Rural	2,548	2,345	9,325
Total	8,489	8,025	16,644

### Prevalence of overweight/obesity

**Figure 1:**
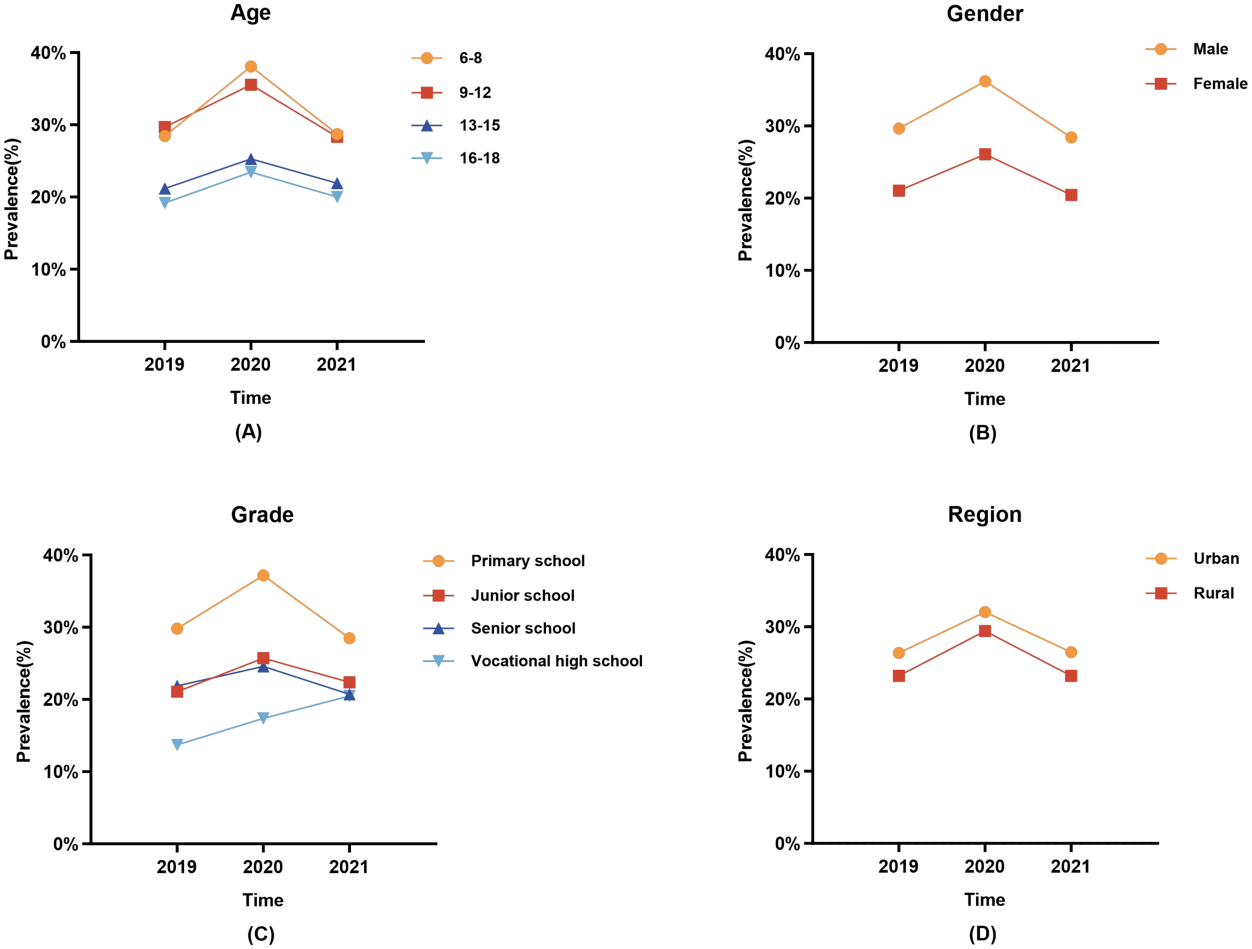
Trends in the prevalence of overweight and obesity among children and adolescents in different subgroups from 2019 to 2021. **(A)** Age; **(B)** Gender; **(C)** Grade; **(D)** Region.

The overall prevalence of overweight/obesity increased from 2019 to 2020, but then decreased in 2021 compared with 2020 (2019, 25.43%; 2020, 31.28%; 2021, 24.64%; *p* < 0.05). Over the 3 years, the total prevalence of overweight/obesity decreased by grade (2019, 29.80 to 13.71%; 2020, 37.18 to 17.37%; 2021, 28.48 to 20.48%; *p* < 0.05). The prevalence of overweight/obesity in boys was higher than in girls (2019, 29.66% vs. 21.04%; 2020, 36.17% vs. 26.07%; 2021, 28.40% vs. 23.20%; *p* < 0.05), and higher in urban areas than in rural areas (2019, 26.38% vs. 23.23%; 2020, 32.04% vs. 29.42%; 2021, 26.48% vs. 23.20%; *p* < 0.05). Moreover, the prevalence of overweight/obesity in the 16–18 age group was significantly lower than in other age groups (2019, 19.19%; 2020, 23.45%; 2021, 20.03%, *p* < 0.05) (Table 2 and Figure 1).

**Table 2:** Prevalence of overweight/obesity among children and adolescents.

Variables	2019	2020	2021	*P* ^a^	*P* ^b^	*P* ^c^
Gender						
Male	29.66	36.17	28.40	<0.05	<0.05	0.135
Female	21.04	26.07	20.44	<0.05	<0.05	0.439
Age						
6–8	28.44	38.09	28.73	<0.05	<0.05	0.834
9–12	29.69	35.54	28.34	<0.05	<0.05	0.193
13–15	21.19	25.27	21.90	<0.05	<0.05	0.515
16–18	19.19	23.45	20.03	<0.05	<0.05	0.487
Grade						
Primary school	29.80	37.18	28.48	<0.05	<0.05	0.125
Junior school	21.09	25.71	22.39	<0.05	<0.05	0.231
Senior school	21.88	24.58	20.71	0.092	<0.05	0.377
Vocational high school	13.71	17.37	20.48	0.235	0.258	<0.05
Region						
Urban	26.38	32.04	26.48	<0.05	<0.05	0.894
Rural	23.23	29.42	23.20	<0.05	<0.05	0.968
Total	25.43	31.28	24.64	<0.05	<0.05	0.169

a, b and c represent *P*-values for multiple comparisons between 2019 and 2020, 2020 and 2021, 2019 and 2021, respectively.

### Prevalence of poor vision

**Figure 2:**
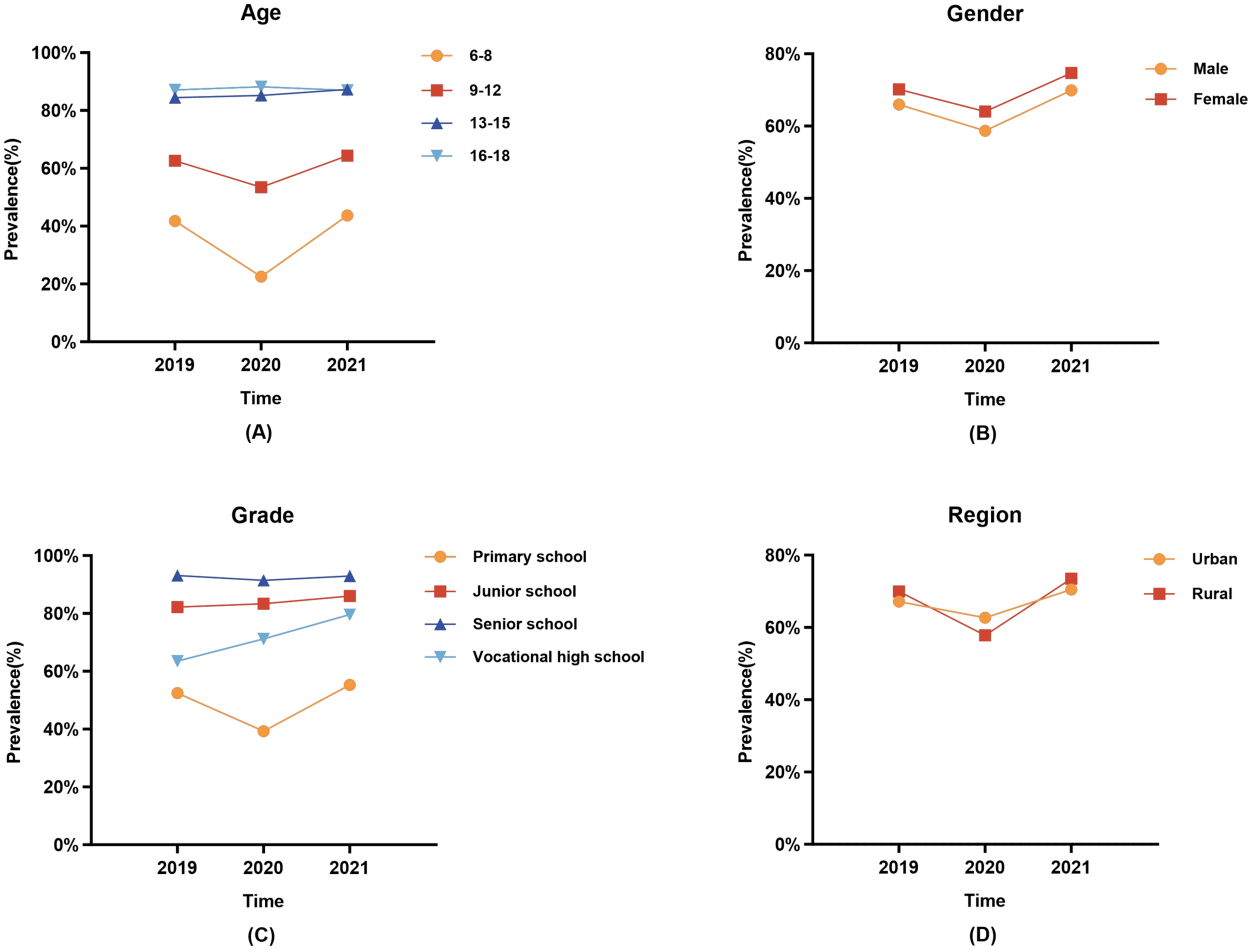
Trends in the prevalence of poor vision among children and adolescents in different subgroups from 2019 to 2021. **(A)** Age; **(B)** Gender; **(C)** Grade; **(D)** Region.

The prevalence of poor vision was 68.02% in 2019, decreased to 61.30% in 2020, and increased to 72.18% in 2021 (*p* < 0.05). The prevalence of poor vision is higher among girls than boys (2019, 70.14% vs. 65.97%; 2020, 64.03% vs. 58.73%; 2021, 74.73% vs. 69.89%; *p* < 0.05), with the highest proportion observed among high school students (2019, 93.13%; 2020, 91.44%; 2021, 92.93%; *p* < 0.05). Furthermore, except for the year 2020, the prevalence of poor vision among students in rural areas exceeded that of urban areas in both 2019 and 2021 (2019, 69.94% vs. 67.19%; 2020, 57.83% vs. 62.73%; 2021, 73.50% vs. 70.49%; *p* < 0.05) (Table 3 and Figure 2).

**Table 3:** Prevalence of poor vision among children and adolescents.

Variables	2019	2020	2021	*P^a^*	*P^b^*	*P^c^*
Gender						
Male	65.97	58.73	69.89	< 0.05	< 0.05	< 0.05
Female	70.14	64.03	74.73	< 0.05	< 0.05	< 0.05
Age						
6–8	41.78	22.53	43.70	< 0.05	< 0.05	0.192
9–12	62.59	53.48	64.34	< 0.05	< 0.05	0.111
13–15	84.43	85.24	87.32	0.457	< 0.05	< 0.05
16–18	87.14	88.20	86.92	0.389	0.213	0.831
Grade						
Primary school	52.49	39.28	55.28	< 0.05	< 0.05	< 0.05
Junior school	82.21	83.39	86.06	0.29	< 0.05	< 0.05
Senior school	93.13	91.44	92.93	0.095	0.086	< 0.05
Vocational high school	63.55	71.19	79.61	0.059	< 0.05	< 0.05
Region						
Urban	67.19	62.73	70.49	< 0.05	< 0.05	< 0.05
Rural	69.94	57.83	73.50	< 0.05	< 0.05	< 0.05
Total	68.02	61.30	72.18	< 0.05	< 0.05	< 0.05

a, b, and c represent *P*-values for multiple comparisons between 2019 and 2020, 2020 and 2021, 2019 and 2021, respectively.

### Characteristics associated with overweight/obesity

The analysis revealed that gender, region, frequency of fruit consumption, smoking status, and physical activity significantly impacted the prevalence of overweight/obesity. Factors associated with a higher prevalence included consuming fresh fruit more than twice daily, non-smoking behavior, engaging in 1–3 h of moderate-intensity exercise per day, and participating in outdoor sports for 3 h or more (Table 4).

**Table 4:** Factors associated with overweight/obesity in children and adolescents.

Factor	Type	Prevalence (%)	Univariate logistic regression	Multivariate logistic regression
			*χ^2^*	OR (95%CI)
Gender	Male	47.39	294.52*	Reference
	Female	34.25		0.596(0.563–0.631)*
Region	Urban	43.65	21.016*	1
	Rural	32.47		0.851(0.804–0.901)*
Eat sweets	Never	26.17	4.19	–
	<1/d	25.02		
	>1/d	26.51		
Eat fried food	Never	25.90	1.37	–
	<1/d	25.16		
	>1/d	25.57		
Eat fresh fruit	Never	24.82	106.22*	Reference
	<1/d	22.02		0.847(0.739–0.971)*
	1/d	26.45		1.048(0.914–1.201)
	>1/d	29.40		1.194(1.036–1.376)*
Frequency of breakfast	Never	26.06	1.996	–
	Sometimes	24.55		
	Every day	25.54		
Smoking cigarette	Yes	24.88	14.19*	Reference
	No	27.63		1.153(1.025–1.297)*
Moderate to high intensity exercise time per week	Never	22.54	35.93*	Reference
	1–3 d	25.97		1.124(1.03–1.227)*
	4–6 d	25.26		1.054(0.956–1.162)
	7 d	26.70		1.088(0.976–1.212)
Daytime outdoor activity hours	<1 h	23.24	25.238*	Reference
	1–2 h	25.50		1.075(0.993–1.165)
	2-3 h	25.68		1.073(0.975–1.18)
	>3 h	27.52		1.178(1.074–1.292)*
	Do not know	24.64		1.065(0.928–1.222)

^∗^Significance is declared at *p* < 0.05.

### Characteristics associated with poor vision

Logistic regression analysis indicated a negative association between living in rural areas, practicing eye exercises, and participating in outdoor activities with the risk of poor vision. Conversely, factors such as male sex, turn off the lights when looking at electronic screens after dark, reading books or electronic screens while lying down, reading books or electronic screens when walking or riding in a car, outdoor activity time, and parental myopia were associated with an increased risk of poor vision (Table 5).

**Table 5:** Factors associated with poor vision in children and adolescents.

Factor	Type	Prevalence (%)	Univariate logistic regression	Multivariate logistic regression
			*χ^2^*	OR (95%CI)
Gender	Male	74.28	125.5*	Reference
	Female	79.99		1.52(1.429–1.617)*
Region	Urban	78.02	21.091*	Reference
	Rural	76.3		0.939(0.882–1)*
Frequency of seat changes	Never	73.29	487.33	–
	Once a term	72.92		
	Once a month	86.38		
	Biweekly	80.56		
	Once a week	71.8		
Frequency of daily eye exercise	Once	80.12	454.63*	Reference
	Twice	71.24		0.851(0.79–0.916)*
	Three times or more	74.41		0.867(0.777–0.967)*
	Never	86.98		1.317(1.175–1.476)*
Location of recess activities	Inside the school building	78.88	232.21*	Reference
	Indoor	68.27		0.753(0.697–0.812)*
Turn off the lights when looking at electronic screens after dark	Never	72.21	317.84*	Reference
	Occasionally	81.48		1.135(1.047–1.232)*
	Frequently	83.07		1.048(0.93–1.181)
	Always	77.89		0.883(0.76–1.026)
Reading books or electronic screens while lying down	Never	70.39	388.24*	Reference
	Occasionally	79.52		1.132(1.047–1.225)*
	Frequently	83.62		1.241(1.106–1.393)*
	Always	82.11		1.274(1.023–1.585)*
Reading books or electronic screens while walking or riding in a car	Never	72.36	364.63*	Reference
	Occasionally	81.68		1.107(1.022–1.2)*
	Frequently	85.17		1.186(1.031–1.364)*
	Always	80.48		0.93(0.708–1.223)
Daytime outdoor activity hours	<1 h	79.91	79.22*	Reference
	1–2 h	77.36		0.99(0.907–1.081)
	2–3 h	76.94		0.959(0.865–1.063)
	>3 h	72.57		0.776(0.703–0.856)*
	Do not know	78.16		0.964(0.828–1.122)
Parental myopia status	Neither parent is myopic	74.53	134.54*	Reference
	Mother is myopic	79.45		2.05(1.49–2.82)*
	Father is myopic	82.95		2.01(1.52–2.66)*
	Both parents are myopic	80.13		2.332(2.074–2.514)*

^*^Significance is declared at *p* < 0.05.

## Discussion

This study investigates the health issues of children and adolescents in a city in Sichuan Province, China, aiming to examine changes in the prevalence of overweight/obesity and poor vision among this population over 3 years during the COVID-19 pandemic, as well as to explore influencing factors. The findings reveal that the prevalence of overweight/obesity was 25.43% in 2019, increased to 31.28% in 2020, and then decreased to 24.64% in 2021. Additionally, the prevalence of poor vision decreased from 68.02% in 2019 to 61.03% in 2020, but subsequently increased to 72.18% in 2021. The contributing factors to overweight/obesity and poor vision may encompass dietary habits, physical activity levels, and lifestyle patterns, among others.

Our research findings indicate a 5.83% increase in the prevalence of overweight/obesity among children and adolescents following the COVID-19 outbreak, aligning with similar trends observed in other countries such as the United States (10), South Korea (11), and Croatia (12). A meta-analysis has revealed that physical inactivity, sedentary lifestyles, and poor dietary patterns are prevalent risk factors for obesity during the COVID-19 pandemic (13). The heightened prevalence of overweight/obesity among children and adolescents is attributed to prolonged school closures and home quarantine during the COVID-19 pandemic, leading to reduced physical activity, increased sedentary behavior, unhealthy eating habits, and elevated mental stress levels. Our observations also indicate a declining trend in overweight/obesity prevalence in 2021, consistent with findings from the Jinan study (14). The resumption of offline school instruction in 2021 has enabled children and adolescents to engage in physical education classes and other outdoor activities, providing more opportunities for physical activity and thereby leading to a decrease in overweight/obesity prevalence.

Regarding the influencing factors of overweight/obesity, the study identified gender as a factor associated with overweight and obesity in children and adolescents, with boys showing a higher likelihood of being overweight or obese than girls. Research involving Chinese children aged 7–12 demonstrated that boys were more likely to have increased body mass index during the period of school closure with COVID-19 (15). Similarly, a simulation study on the impact of COVID-19 on obesity and body mass index in American children indicated a greater effect on boys compared to girls (16). On the one hand, boys tend to spend more time on physical activity than girls. However, during COVID-19 home closure and school closure, boys showed reduced physical activity and increased sedentary behavior (17). On the other hand, girls ate more vegetables and fruits during the epidemic, while boys increased their intake of processed meats due to differences in food preferences and serving sizes between men and women (18). These factors may have contributed to quicker weight gain in boys than girls during the COVID-19 pandemic, consequently elevating the risk of overweight and obesity. In terms of regional disparities, students residing in urban areas exhibited a heightened susceptibility to overweight and obesity compared to their rural counterparts, aligning with findings from prior scholarly investigations (19, 20).

Additionally, we found that individuals who consumed fresh fruits more than twice a day, did not smoke, engaged in 1–3 days of moderate to high-intensity exercise, and spent 3 or more hours on outdoor activities daily were at a higher risk of overweight and obesity, contradicting conventional wisdom. This unexpected finding may stem from the survey’s lack of information on students’ daily dietary intake, family economic status, and parents’ knowledge of nutrition and health, introducing a potential bias.

This study employed the standard logarithmic visual acuity chart to assess poor vision, a widely adopted method for large – scale investigations. However, as a purely behavioral assessment, its results are susceptible to subjective factors of the participants. Recently, objective and non-invasive techniques for detecting early visual impairment have gained increasing attention. One such technique is visual evoked potentials (VEPs), which measure the cortical responses triggered by visual stimuli and can be recorded non-invasively through electrodes placed on the scalp. Clinically, VEPs are utilized to evaluate and characterize the functions of normal and abnormal visual pathways, providing objective diagnostic information (21, 22). Notably, pattern reversal visual evoked potentials (PRVEPs) have demonstrated significant prognostic value for the future visual development of young children with cerebral visual impairment (23). These advanced techniques open up promising avenues for future research on visual health in children and adolescents.

Our study revealed a fluctuating trend in the overall prevalence of poor vision among children and adolescents in the target city from 2019 to 2021. The prevalence initially decreased from 2019 to 2020 but rebounded in 2021. At the onset of the COVID-19 pandemic, school closures likely alleviated students’ academic workloads, allowing their eyes more time to rest and recover, which contributed to the decline in poor vision rates. Additionally, during the period of online learning at home, parental supervision facilitated the correction of children’s improper eye – use habits, further preventing poor vision. However, in 2021, despite the gradual resumption of in – person schooling, sporadic local outbreaks led to irregular class suspensions and a return to online courses. This shift resulted in an increase in the prevalence of poor vision from 61.03 to 72.18%, indicating that the pandemic had a substantial impact on the visual development of children and adolescents and potentially increased the burden on healthcare systems for managing poor vision and myopia. Furthermore, our data showed that girls had a higher prevalence of poor vision compared to boys. This gender disparity may be attributed to girls’ tendency to spend more time on reading and close – work activities and less time on outdoor activities (24, 25). Students in urban areas were also more prone to poor vision than their rural counterparts. As suggested by Guo et al. (26), urban students typically engage in less outdoor activity and spend extended periods watching television. Abundant evidence from previous studies (27–29) has demonstrated the protective effect of outdoor activities on visual health. Outdoor exposure promotes the synthesis of vitamin D in the body, and adequate vitamin D levels have been associated with a reduced risk of myopia (30).

The visual development of children and adolescents is a dynamic process. During this period, environmental factors such as screen exposure and light can have disproportionate impacts on children and adolescents of different age groups. The critical period of human visual development is within the first 24 months after birth, and the sensitive period can last until the age of 9–12 (31). For children and adolescents in the sensitive period, their visual systems are still continuously developing and improving, making them more sensitive to the influences of environmental factors. Barlow (32) proposed that the human visual processing system is specifically designed to effectively encode various images that we commonly encounter in our daily environment. Visual discomfort or fatigue may be associated with the overrepresentation of certain spatial frequencies. Visual behaviors like excessive screen use can lead to excessive stimulation of specific spatial frequencies, triggering non-optimal encoding in the visual system, which in turn increases visual discomfort and fatigue (33, 34). In this study, behaviors such as reading while lying down, using electronic devices, watching screens while walking, or viewing screens in moving vehicles are risk factors for poor vision, which is consistent with the conclusions pointed out by Wang et al. (35) and Shi et al. (36). These behaviors can increase accommodative tension and cause extraocular muscle imbalance, thereby exacerbating neuro-ophthalmic fatigue. In addition to the impacts of improper behavioral postures, environmental factors also come into play. Under conditions of low illumination and darkness, the eye axis will elongate, which can give rise to myopia (37).

The development of poor vision is influenced not only by external environmental factors, but also by genetic factors. Previous studies have indicated that parental myopia plays a significant role in the occurrence of myopia in children and adolescents (38–42). In this study, students with only a father with myopia, only a mother with myopia, and both parents with myopia had 2.01, 2.05, and 2.332 times higher risk of developing poor vision, respectively, compared to students whose parents did not have myopia. In conclusion, poor vision is associated with several factors such as gender, behavioral habits, and environment, suggesting the need for a comprehensive prevention strategy to enhance eye health from multiple perspectives.

There are some limitations to this study. Firstly, due to the limited literacy skills of school-aged children aged 6–8 years who could not complete the questionnaire, only the responses from students aged 9–18 were included for the analysis of influencing factors. Secondly, as a single – center investigation, the study’s environment might have been influenced by specific research conditions, operational procedures, and the researchers’ expertise, potentially introducing selection and information bias. Extrapolation of the findings to other regions or populations should therefore be approached with caution. Lastly, information on additional risk factors for overweight, obesity, and poor vision, including daily dietary habits, family economic status, parental nutrition and health awareness, as well as screen time and sleep duration, was not collected in this survey, potentially introducing bias to the results.

## Conclusion

The COVID-19 pandemic has had profound implications for the physical and mental well-being of children and adolescents, resulting in noticeable fluctuations in rates of overweight/obesity and poor vision before and after the outbreak of the pandemic. Therefore, strategies for the prevention and management of common childhood and adolescent diseases should be rigorously developed to ensure that children and adolescents are able to develop fully and remain physically and mentally healthy.

## Data Availability

The datasets presented in this article are not readily available because the data utilized in this study was sourced from the non-public database of Mianyang Center for Disease Control and Prevention. Requests to access the datasets should be directed to Nana Li, 1561636900@qq.com.
